# Tophaceous gout of the nose in a male *FMR1* premutation carrier

**DOI:** 10.1002/ccr3.6586

**Published:** 2022-11-27

**Authors:** Si Jie Tang, Shanthi Giri, Nima Pahlavan, Sophia H. Han, Ellery R. Santos, Glenda Espinal, Ramkumar Aishworiya, Andrea Schneider, David Hessl, Susan M. Rivera, Randi J. Hagerman

**Affiliations:** ^1^ Medical Investigation of Neurodevelopmental Disorders (MIND) Institute University of California Davis Davis California USA; ^2^ Kaiser Permanente Sacramento California USA; ^3^ Kaiser Permanente North Valley Roseville California USA; ^4^ Mira Loma High School Sacramento California USA; ^5^ Khoo Teck Puat‐National University Children's Medical Institute National University Health System Singapore Singapore; ^6^ Department of Pediatrics, Yong Loo Lin School of Medicine National University of Singapore Singapore Singapore; ^7^ Department of Psychiatry and Behavioral Sciences University of California Davis School of Medicine Sacramento California USA; ^8^ University of Maryland College Park Maryland USA; ^9^ Department of Pediatrics University of California Davis School of Medicine Sacramento California USA

**Keywords:** *FMR1*, FXTAS, gout, premutation, tophi

## Abstract

Premutation alleles with 55–200 CGG repeats in *FMR1* can lead to fragile X‐associated tremor/ataxia syndrome (FXTAS). In this case study, we report uncontrolled gout in a 68‐year‐old male with FXTAS with multiple sites of involvement including a rare gouty tophus in the nasal region.

## Graphical abstract

This is the first documentation of immune dysregulation manifesting as uncontrolled gout in a male FXTAS premutation carrier. There is a need for further investigation of immune‐mediated disorders in male FXTAS carriers.

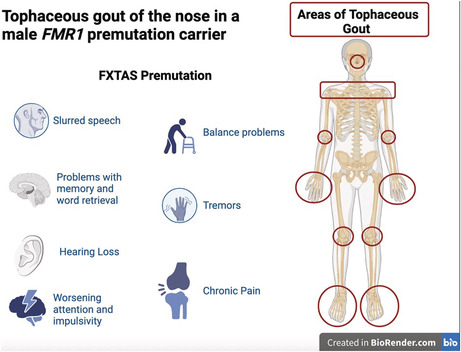

## 1 INTRODUCTION

Fragile X‐associated tremor/ataxia syndrome (FXTAS; OMIM 300623) is an adult‐onset neurodegenerative disorder that is characterized by intention tremor, cerebellar ataxia, neuropathy, memory deficits, parkinsonian features, central nervous system (CNS) atrophy, and white matter disease.^1^ FXTAS is one of the disorders associated with the fragile X premutation which is characterized by the expansion of the non‐coding CGG repeat region (55–200 repeats) of the fragile X messenger ribonucleoprotein 1 gene (*FMR1*). The premutation causes elevated levels of *FMR1* mRNA and a subsequent gain‐of‐function toxicity resulting in the formation of intranuclear inclusions in neurons and astrocytes in numerous brain regions and in the peripheral nervous system.^2^

Immune‐mediated disorders, such as autoimmune thyroid disease and fibromyalgia, have been observed to be significantly increased in women with FXTAS.^3^, ^4^ Currently, there are no reports of immune‐mediated disorders that are common in males with FXTAS. One form of immune dysregulation is gout, which is the most common form of inflammatory arthritis with a prevalence ranging from <1% to 6.8% globally.^5^ The occurrence is highest in developed countries such as the United States, with a prevalence of 3.9% among all adults.^6^ Acute and chronic gout can lead to a decrease in the quality of life.^7^ In 2015–2016, the average age of patients with gout was 48 years old and was observed to be more common in men than women.^6^ Gout presents clinically with episodes of intense joint pain followed by symptom‐free periods.

Hyperuricemia is a risk factor of gout, leading to the formation of monosodium urate (MSU) crystals deposited at joints which induce an inflammatory response in the body.^8^ Urate is a product of purine mononucleotide degradation in the body and is excreted out of the body through urate transporters in the proximal tubule epithelial cells. When urate clearance in the kidneys is diminished, saturation of urate in the blood occurs which increases the deposition of urate into crystals. The inflammatory process of gout is mediated by the phagocytosis of MSU crystals and activation of the NLRP2 and NLRP3 inflammasome.^8^, ^9^ Proinflammatory cytokines and interleukin 1β amplify the inflammatory process and recruit neutrophils, monocytes, macrophages, and lymphocytes.^8^

When gout is uncontrolled, urate crystals are deposited in joints as tophi.^10^ Tophi are commonly found on the knees, toes, wrists, and fingers, but rarely in the nasal area.^11^ In this case report, we describe a 68‐year‐old male with FXTAS who presents with a rare gouty tophus in the nasal region and at multiple additional locations.

## 2 CASE PRESENTATION

A 68‐year‐old male with the fragile X premutation (59 CGG repeats) developed intermittent tremor at age 55 and mild balance problems at age 59. These symptoms gradually worsened over time. He also had a history of anxiety and attention‐deficit hyperactivity disorder (ADHD) since childhood, reclusive behavior in high school, and difficulty with making direct eye contact with people. He developed hypertension at age 56; self‐reported daily memory problems which began at age 57; swallowing problems at age 59; and had insomnia for years but sleep apnea was diagnosed at age 61 and he was treated with continuous positive airway pressure (CPAP) therapy. He had depression in the past and currently still has social phobia, generalized for most social situations. He has had symptoms of chronic pain because of neuropathy in his legs and has been taking MS Contin (Morphine) for chronic back pain. He had complained of chronic fatigue over the last few years.

**FIGURE 1:**
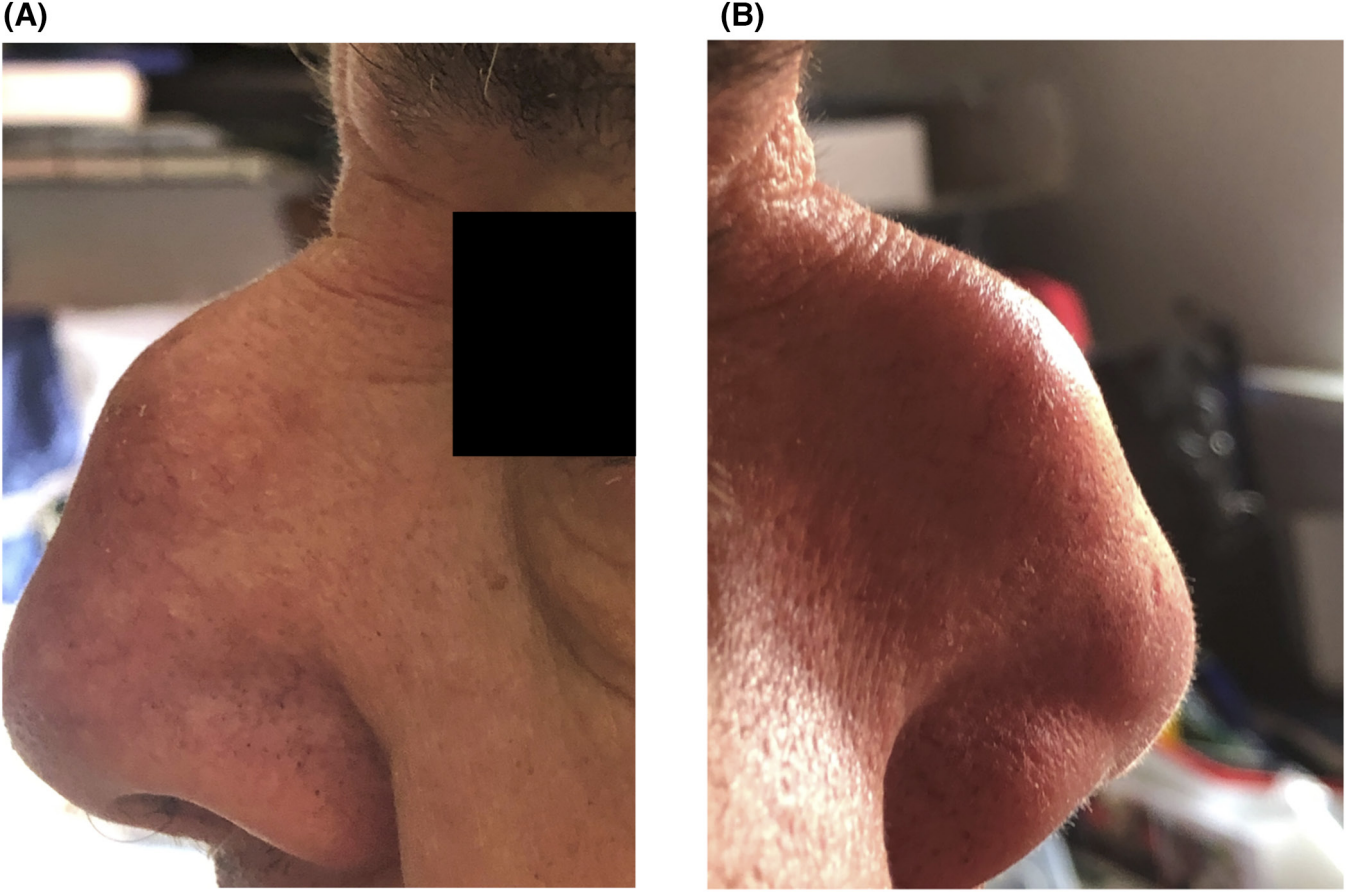
Presurgical image of tophaceous gout in the nasal region. (A) Sagittal view of left side. (B) Sagittal view of right side.

**FIGURE 2:**
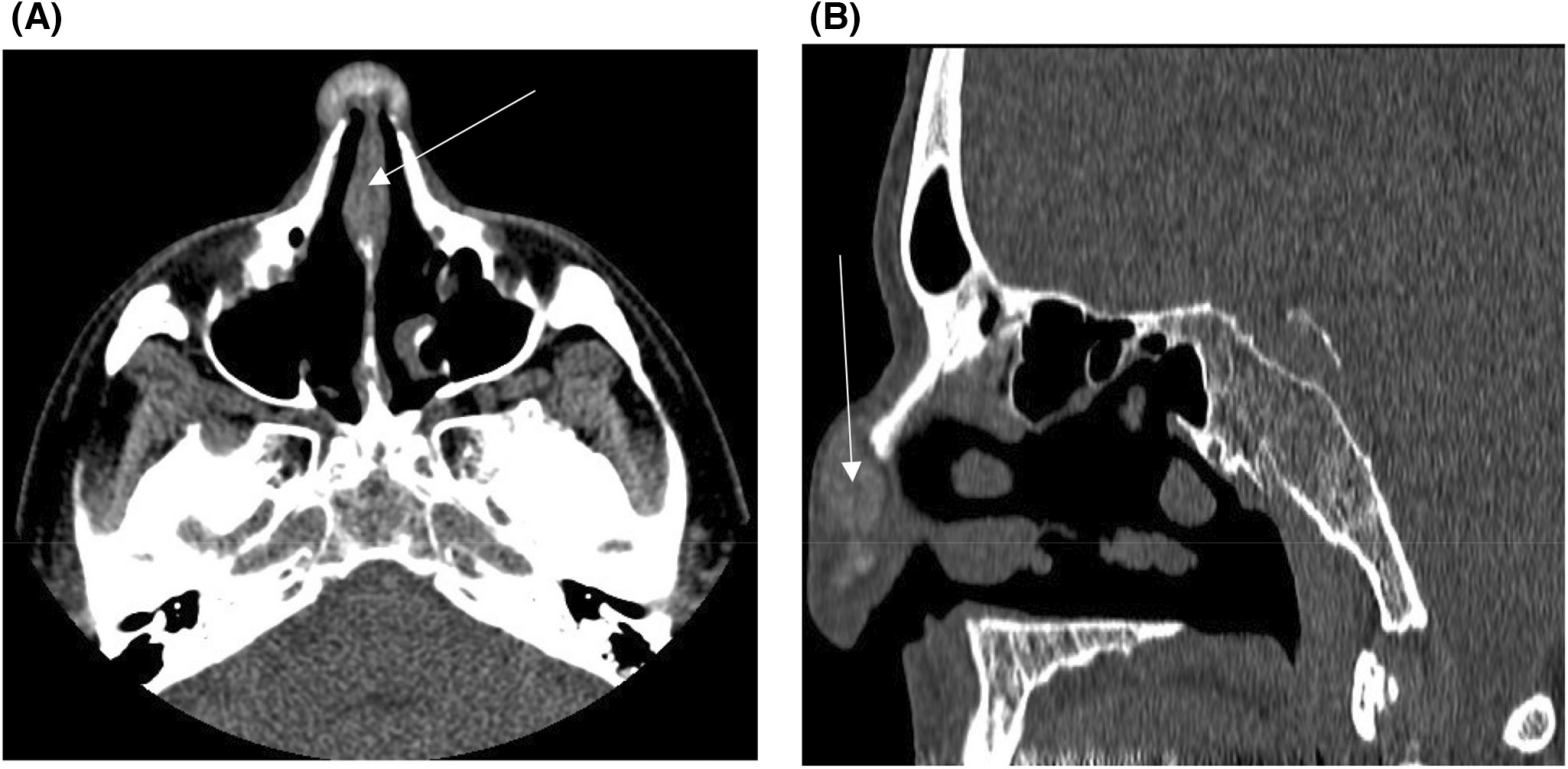
CT scan showing nodular soft tissue lesion in the nose and histological features of nasal tophi. (A) Axial view. (B) Lateral view. Arrows indicate tophus.

**FIGURE 3:**
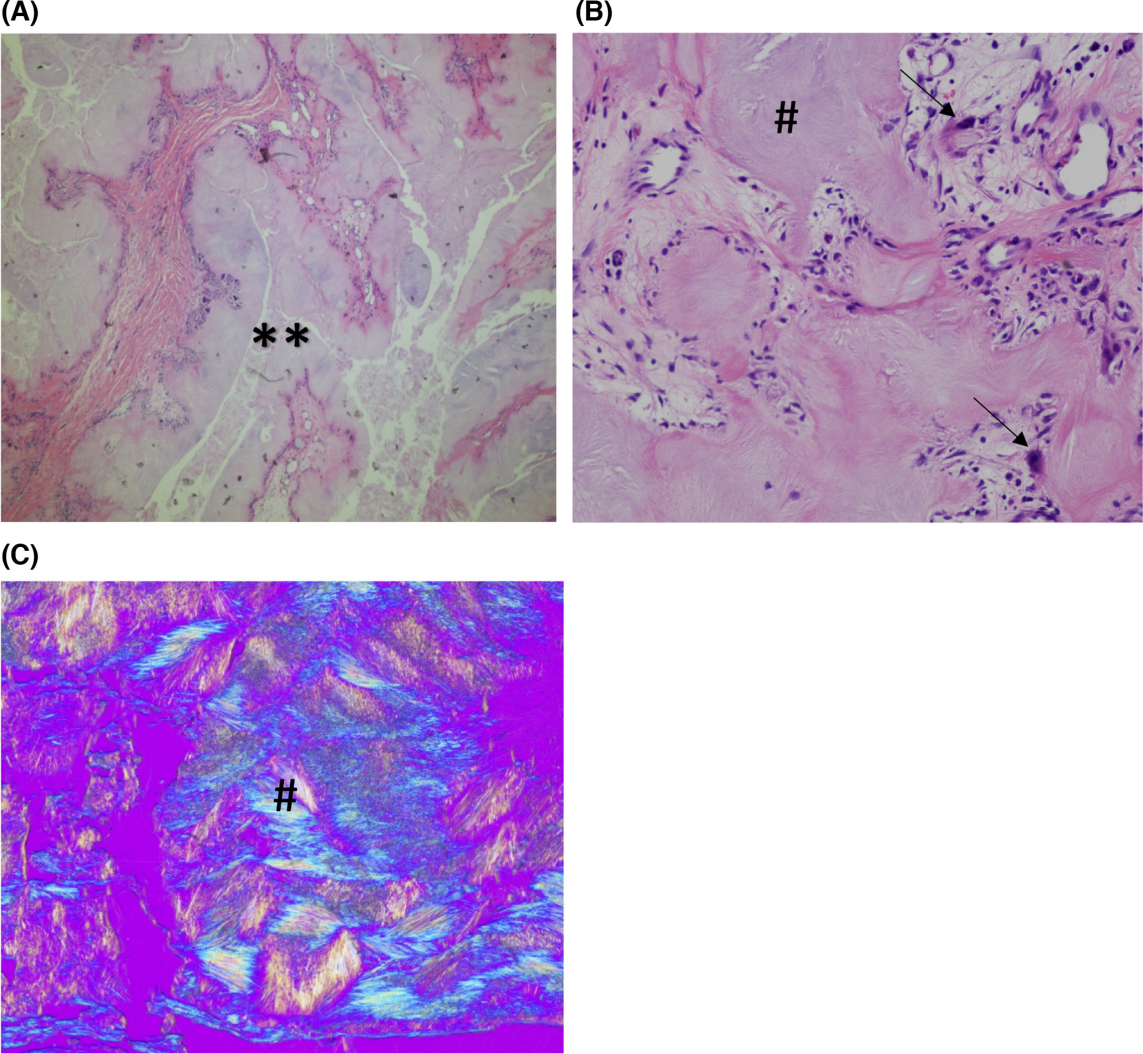
(A) Low‐power view of gouty tophi (** indicating tophi) appear as large amorphous deposits in subcutaneous tissues surrounded by granulomatous inflammation and foreign body giant cells [hematoxylin and eosin (HE), 40×]. (B) high‐power magnification showing the deposited MSU crystals (# indicating the crystals) surrounded by an inflammatory reaction consisting of foreign body giant cells (arrows indicating giant cells), macrophages, and lymphocytes. (H&E, 200×). (C) MSU crystals (# indicating the crystals) are needle‐shaped and exhibit negative birefringence when examined with a polarizing filter and red compensator filter.

His gout began at age 54 and has spread dramatically throughout his body including tophi on his nose, clavicles, elbows, knees, fingers, and toes. He had tophaceous gout in his nasal area which was surgically removed (Figure 1A,B). A computed tomographic (CT) scan showed nodular soft tissue lesion with partial calcification in the nose, measuring approximately 2.8 cm by 1.5 cm by 1.8 cm (Figure 2A,B). A pathology report confirmed that it was a gouty tophus with a granuloma. Histologically, the MSU crystals were surrounded by chronic mononuclear and giant cell reactions in soft tissue (Figure 3A,B). MSU crystals are needle‐shaped and exhibit negative birefringence when examined with a polarizing filter and red compensator filter (Figure 3C).

He was prescribed allopurinol 400 mg daily to treat hyperuricemia and colchicine 0.6 mg twice daily as a prophylaxis for gout due to recurrent flareups. Patient was noncompliant with treatment–allopurinol and colchicine were taken intermittently, and he was treated with short courses of prednisone for acute episodes. He had developed tophi in multiple areas also likely due to poor compliance. The patient had been seen by a rheumatologist from 2013 to 2021 when he had gout flares in the sternoclavicular joints without any tophi. His uric acid remained elevated in the range of 7.1–11.9 mg/dl from 2016 to 2021. It was normal (5.0) for the first time in September 1, 2021.

On examination in April 2022, his vital signs included a blood pressure of 140/95 mm Hg, pulse of 64 beats/minute, BMI 25.3, height of 188.1 cm, and weight of 89.5 kg. Tophi were observed throughout his body, including on his clavicles bilaterally, on his hands and finger joints, on his elbow, severely on his knees and feet, and on his toes bilaterally. His muscle strength was normal although power on the left leg and left arm were somewhat weaker than the right side, but he also had a torn rotator cuff on the right arm. His deep tendon reflexes were hypoactive in the upper extremities, 1+ in knees, and absent in the ankles. The Babinski reflex was equivocal, but he had plantar fasciitis in the right foot. His temperature sensation was absent in the feet, and his vibration sensation was absent on the left side and significantly diminished on the right. He had mild swelling in his feet. His pinprick sensation was decreased bilaterally and there were some parts of his feet bilaterally that are completely numb, particularly the toes. His tremor was very minimal with finger‐to‐nose testing and he had a slight positional tremor, but no resting tremor was seen. He did have cerebellar ataxia with heel‐to‐shin movements and his tandem walking was difficult and he was only able to take three to four steps.

The patient presented to the surgeon with an inflammatory lesion over the nasal dorsum. A direct incision was created over the patient's nasal dorsum in the shape of a hockey stick with the apex of the incision superiorly in the region of the glabella. A skin flap was elevated, and the lesion was excised via sharp and blunt dissection. After obtaining hemostasis, the incision was closed, and no nasal reconstruction was conducted. Six months after the operation, the patient did not have any nasal lesion or mass on physical examination during clinical follow‐up.

## 3 DISCUSSION

This is the first reported case of nasal gout associated with the immune deficits of a male with FXTAS. His symptoms of hypertension, insomnia, chronic pain, and chronic fatigue are generally associated with the premutation, and his symptoms of tremor, balance problems, slurred speech, significant hearing loss, worsening of his attention, and severe problems with memory and word retrieval are all associated with his diagnosis of FXTAS.^12^ Genetic counseling was provided to the patient. He has a daughter with the premutation, but she has the fragile X‐associated neuropsychiatric disorder (FXAND) including emotional problems and chronic drug abuse. His granddaughter also has the premutation and has mild developmental delays.

Gouty tophi are features of advanced gout.^13^ Gout deposits in the nasal region are quite rare with only 10 cases reported in literature.^14^ Previous reports in literature of patients with nasal gout were in middle‐aged men with a history of gouty arthritis. Here, we present a patient who matches this demographic except at a relatively older age. There is reason to believe that his uncontrolled gout could be due to the immune deficits due to his FXTAS.^3^, ^4^ Given the late onset of gout in this patient, it is unlikely that it could be due to other genetic mutations that are canonically associated with gout. Mutations of the enzyme hypoxanthine‐guanine phosphoribosyl transferase (HPRT) for purine salvage or the hyperactivity of enzyme 5‐phosphoribosyl‐1‐pyrophospate (PRPP) synthetase as a co‐substrate for HPRT usually lead to gouty arthritis during childhood and early adulthood.^15^

We postulate that males with FXTAS may have an increased prevalence of immune‐mediated disorders just as observed in females with FXTAS.^3^, ^4^ In fibroblasts and brain samples from premutation carriers, there is evidence of mitochondria dysfunction.^16^ Similarly, mitochondrial stress has been shown to induce NLRP3 inflammasome activation leading to many chronic diseases, including gout.^17^ Therefore, there is reason to believe that the mitochondria dysregulation due to elevated levels of *FMR1*‐mRNA can lead to inflammasome activation manifesting into gout.

Another possible mechanism by which the premutation may lead to immune dysregulation is through the secondary effects of toxicity associated with excessive mRNA which can sequester proteins and dysregulate miRNAs.^4^, ^18^ miRNAs have been shown to play an important role in the development and maintenance of the immune system.^19^ In a study of plasma from patients with gout, one of the miRNAs that was upregulated was hsa‐miR‐142‐3p.^20^ Similarly, hsa‐miR‐142‐3p was also found to be dysregulated in the blood of FXTAS patients.^21^ The theory of the premutation leading to immune dysfunction and gout is supported by the observation that the peripheral blood of males with FXTAS have elevated IL‐10 a proinflammatory cytokine which is a mediator in the development of gout.^18^, ^22^ Therefore, it is possible that the presence of a premutation leads to immune dysfunction and in the patient described here, an exacerbation of poorly controlled gout. Toxicity related to elevated mRNA levels has already been shown to be a crucial mechanism of many other disorders related to the premutation state, including FXTAS, fragile X‐associated primary ovarian insufficiency (FXPOI; OMIM 311360), and fragile X‐ associated neuropsychiatric disorders (FXAND).^23^ Immune dysregulation could perhaps be another consequence of this toxicity, although further research on the exact underlying mechanisms is warranted.

In this case study, we report rare tophaceous gout of the nose, clavicles, and all extremities in a male premutation carrier with FXTAS. This is the first description of an atypical manifestation of uncontrolled gout in a male premutation carrier. A routine uricemia screening would prevent onset in this vulnerable population. Additional investigation of the immune‐mediated disorders of male FXTAS carriers is warranted.

## AUTHOR CONTRIBUTIONS

All authors have approved the final article. Si Jie Tang was involved in writing the original draft, review, and editing. Shanthi Giri and Nima Pahlavan was involved in review and editing. Sophia Han, Glenda Espinal, and Ellery R. Santos were involved in review, editing, and visualization. Ramkumar Aishworiya, David Hessl, Susan M. Rivera, and Andrea Schneider were involved in review and editing. Randi J. Hagerman was involved in conceptualization, review, editing, and supervision.

## FUNDING INFORMATION

This research was funded by NICHD grant HD036071, NINDS grant NS110100, and the MIND Institute IDDRC U50 HD103526.

## CONFLICT OF INTEREST

None of the authors report any conflicts of interest.

## CONSENT

Written informed consent was obtained from the patient to publish this report in accordance with the journal's patient consent policy. The patient has signed consent to release the photographs provided for this case report.

## Data Availability

The data that support the findings of this study are available from the corresponding author upon reasonable request.
